# Case series of four psychiatric patients with copy number variations in the neurexin 1 gene

**DOI:** 10.1002/pcn5.36

**Published:** 2022-09-02

**Authors:** Itaru Kushima, Toshiya Inada, Kazutaka Ohi, Jun Egawa, Norio Ozaki

**Affiliations:** ^1^ Department of Psychiatry Nagoya University Graduate School of Medicine Nagoya Japan; ^2^ Medical Genomics Center Nagoya University Hospital Nagoya Japan; ^3^ Department of Psychiatry Gifu University Graduate School of Medicine Gifu Japan; ^4^ Department of General Internal Medicine Kanazawa Medical University Ishikawa Japan; ^5^ Department of Psychiatry Niigata University Graduate School of Medical and Dental Sciences Niigata Japan

## Abstract

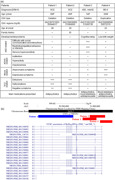

*NRXN1* encodes a presynaptic cell adhesion protein, neurexin 1, and plays an important role in the formation of synapses and release of neurotransmitters.^1^, ^2^ Rare copy number variations (CNVs) of *NRXN1* have been shown to be associated with schizophrenia (SCZ), autism spectrum disorder (ASD), and bipolar disorder (BD).^1^, ^2^, ^3^, ^4^ Here, we report the first case series of four Japanese patients with *NRXN1* CNVs.

These CNVs were identified using array comparative genomic hybridization in our previous studies^5^, ^6^ (see details in the Supporting Information). This study was approved by the ethics committee of Nagoya University. Written informed consent was obtained from participants.

Figure 1A summarizes the clinical data of the four patients with *NRXN1* CNVs. Patient 1 was a 69‐year‐old female with treatment‐resistant SCZ (TRS). Her developmental milestones were unremarkable. She graduated from junior high school with an average grade and moved from one job to another. At 28 years of age, she developed bizarre delusions and auditory hallucinations. She was treated with antipsychotics, but experienced repeated relapses. Over time, her cognitive function deteriorated, she lost her ability to live independently, and eventually she required long‐term hospitalization. At 69 years of age, the delusions and hallucinations persisted despite treatment with high doses of antipsychotics (olanzapine 10 mg/day, aripiprazole 24 mg/day, and risperidone 12 mg/day). Patient 2 was a 46‐year‐old female with TRS. She had a family history of intellectual disability in her mother. Her father died when she was 13 years old and she was placed in foster care. After graduating from high school, she worked diligently as a cashier at a store. At 26 years of age, she developed disorganized speech and auditory hallucinations. Thereafter, she had multiple relapses and hospitalizations. Her psychotic symptoms eventually became refractory to high doses of antipsychotics. At the time of this study, auditory hallucinations were not controlled despite treatment with olanzapine 10 mg/day. Patient 3 was a 14‐year‐old girl with ASD and mild intellectual disability. As a preschooler, she could not participate in group activities and tended to play alone. She was only interested in very selected things and was hypersensitive to sounds and smells. Around 12 years of age, she developed obsessive thoughts of harming others and delusions of persecution and observation, accompanied by mood swings. Sertraline, aripiprazole, and valproate were prescribed for these symptoms. Her IQ was 69 at the age of 13 years. Patient 4 was a 30‐year‐old male with BD‐II. He was born at 36 gestational weeks with low birth weight (2250 g). In elementary school, he exhibited ASD‐ and attention‐deficit/hyperactivity disorder (ADHD)‐like symptoms: he was unable to understand social situations, interpreted language literally, and was hypersensitive to sounds and touch. He was forgetful and frequently lost things. At 23 years of age, he suffered from depressive moods and insomnia and was treated at a psychiatric clinic. At 29 years of age, he developed hypomanic symptoms, including elevated mood, talkativeness, and spending sprees, which were improved with lithium. Thereafter, he again became depressed and was hospitalized. At the time of this study, he was stable with lithium 400 mg/day. His IQ was 87.

**Figure 1:**
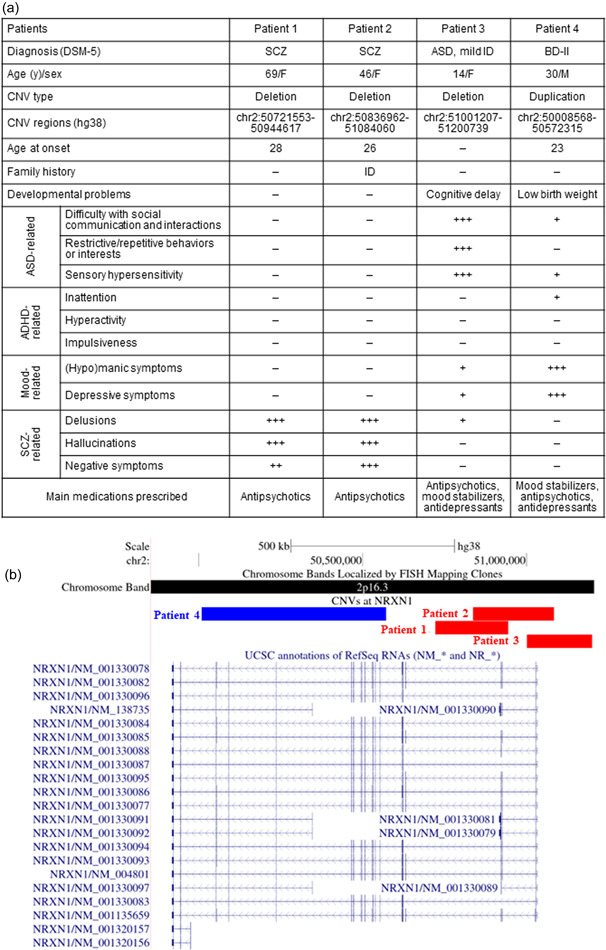
Copy number variations (CNVs) at *NRXN1* and clinical data for the patients. (A) Summary of clinical data of the four patients with CNVs at *NRXN1*. The severity of each psychiatric symptom was graded as one of four levels by board‐certified research psychiatrists: – (none), + (mildly present), ++ (moderately present), and +++ (strongly present). (B) Visualization of CNVs at *NRXN1*. Exonic deletions at the 5' region of *NRXN1* were identified in three patients (Patients 1, 2, and 3). An intragenic duplication was identified in one patient with BD (Patient 4). ADHD, attention‐deficit/hyperactivity disorder; ASD, autism spectrum disorder; BD, bipolar disorder; ID, intellectual disability; SCZ, schizophrenia.

Figure 1B shows *NRXN1* CNVs identified in the four patients. Patients 1–3 had exonic deletions disrupting *NRXN1*. Patient 4 had intragenic duplication, which likely disrupts the transcript reading frame of *NRXN1*. The inheritance pattern was unknown because genomic DNA from parents was not available for any of the patients. None of the patients had any other pathogenic CNVs.

The inter‐individual variability of psychiatric manifestations has been highlighted in patients with *NRXN1* CNVs.^1^, ^2^, ^3^, ^4^ On the other hand, less attention has been paid to the intra‐individual variability of their psychiatric manifestations. The present case series shows not only the inter‐individual variability but also the intra‐individual variability of psychiatric presentations. Patient 3 with ASD and intellectual disability developed psychotic symptoms in her teens. Patient 4 with BD‐II had ASD‐ and ADHD‐like symptoms in his childhood. In addition, two patients with *NRXN1* deletion had TRS. Although there are few case reports of TRS patients with *NRXN1* CNVs, pathogenic CNVs have been reported to be associated with TRS.^5^ Taken together, patients with *NRXN1* CNVs may have more complex and severe psychiatric manifestations. Further case series will be needed to establish the clinical significance of *NRXN1* CNVs.

The limitations of this study are: (1) the retrospective evaluation of phenotypes and (2) the lack of objective assessment of psychiatric symptoms using standardized instruments. Longitudinal, prospective studies with quantitative assessments are needed to further elucidate the natural history of patients with *NRXN*1 CNVs.

## AUTHOR CONTRIBUTIONS

Itaru Kushima designed the study, performed the experiments, analyzed the data, and wrote the first draft of the manuscript. The other authors commented on and refined the manuscript. Toshiya Inada, Kazutaka Ohi, Jun Egawa, and Norio Ozaki recruited the participants and/or collected DNA samples or phenotype data. All authors carefully read the manuscript and approved the final version for submission.

## CONFLICT OF INTEREST

I. K., T. I., K. O., and J. E. declare no conflict of interest. N. O. has received research support or speakers' honoraria from, or has served as a consultant to, Sumitomo Dainippon, Eisai, Otsuka, Kaiteki, Mitsubishi Tanabe, Shionogi, Eli Lilly, Mochida, Daiichi Sankyo, Nihon Medi‐Physics, Takeda, Meiji Seika Pharma, EA Pharma, Pfizer, MSD, Lundbeck Japan, Tsumura, Novartis, Boehringer Ingelheim, Viatris, Kyowa, Janssen, Yoshitomi Yakuhin, Kyowa Kirin, Ono, Astellas, UCB, Taisho Toyama, Medical Review, and Woolsey, outside the submitted work.

## ETHICS APPROVAL STATEMENT

This study was approved by the ethics committee of Nagoya University.

## PATIENT CONSENT STATEMENT

Written informed consent was obtained from all participants.

## CLINICAL TRIAL REGISTRATION

N/A

## Supporting information

Supporting information.

## Data Availability

The data that support the findings of this study are available from the corresponding author upon reasonable request.
