# Supplementary material for: Case series of four psychiatric patients with copy number variations in the neurexin 1 gene
Source: PCN Rep. 2022 Sep 2;1(3):e36. doi: 10.1002/pcn5.36 (PMC11114373; doi:10.1002/pcn5.36)
Supplement: Supplementary file 1 — Supporting information. [file PCN5-1-e36-s001.docx]

**Supplementary materials**

**Case series of four psychiatric patients with copy number variations in the neurexin 1 gene**

Running title: *NRXN1* CNVs in psychiatric disorders

Itaru Kushima*^1,2^, Toshiya Inada^1^, Kazutaka Ohi^3,4^, Jun Egawa^5^, Norio Ozaki^1^

1 Department of Psychiatry, Nagoya University Graduate School of Medicine, Nagoya, Japan

2 Medical Genomics Center, Nagoya University Hospital, Nagoya, Japan

3 Department of Psychiatry, Gifu University Graduate School of Medicine

4 Department of General Internal Medicine, Kanazawa Medical University

5 Department of Psychiatry, Graduate School of Medical and Dental Sciences, Niigata University

**Supplementary Methods**

**Participants**

All patients were of Japanese ancestry. This study was approved by the ethics committee of Nagoya University Graduate School of Medicine and other participating institutes. Written informed consent was obtained from patients or their guardians. Patients were diagnosed according to the Diagnostic and Statistical Manual of Mental Disorders, Fifth Edition criteria for schizophrenia (SCZ), autism spectrum disorder (ASD), and bipolar disorder (BD). The CNVs at *NRXN1* were identified using array comparative genomic hybridization (aCGH) (1, 2).

**Genetic analysis**

Genomic DNA was extracted from blood samples. Two types of aCGH were utilized to identify CNVs at *NRXN1*: NimbleGen 720k Whole-Genome Tiling Arrays (Roche NimbleGen, Madison, WI) (Patient 1), and Agilent SurePrint G3 Human CGH 400k (Agilent, Santa Clara, CA) (Patients 2, 3, and 4). For both types of arrays, we generated CNV calls with Nexus Copy Number software, v9.0 (BioDiscovery, El Segundo, CA) (1). Previously, we confirmed that CNV calls from both types of arrays are highly accurate, with a validation rate of >99%. All genomic locations are given in hg38 coordinates.

**Phenotypic analysis**

We retrospectively collected clinical data of four patients with CNVs at *NRXN1* from their medical records. The data included developmental history, family history, medical history, psychiatric symptoms, age at onset of psychiatric disorders, history of hospitalizations, medications, and treatment response or resistance. Based on the data, the severity of symptoms was graded as one of four levels by board-certified research psychiatrists: – (none), + (mildly present), ++ (moderately present), and +++ (strongly present).

Treatment-resistant schizophrenia (TRS) was defined as the persistence of symptoms despite ≥2 trials of adequate doses (≥600 mg/day chlorpromazine equivalent) and duration (≥4 weeks at a therapeutic dosage) of antipsychotic medications with documented adherence (3).

**References**

1. Kushima I, Aleksic B, Nakatochi M, Shimamura T, Okada T, Uno Y, et al. Comparative Analyses of Copy-Number Variation in Autism Spectrum Disorder and Schizophrenia Reveal Etiological Overlap and Biological Insights. Cell Rep. 2018;24(11):2838-56.

2. Kushima I, Aleksic B, Nakatochi M, Shimamura T, Shiino T, Yoshimi A, et al. High-resolution copy number variation analysis of schizophrenia in Japan. Mol Psychiatry. 2017;22(3):430-40.

3. Howes OD, McCutcheon R, Agid O, de Bartolomeis A, van Beveren NJ, Birnbaum ML, et al. Treatment-Resistant Schizophrenia: Treatment Response and Resistance in Psychosis (TRRIP) Working Group Consensus Guidelines on Diagnosis and Terminology. Am J Psychiatry. 2017;174(3):216-29.
